# Evaluation of Differentiating Attacking and Defensive Performance for Various Playing Positions during the Tokyo Olympics Men's Basketball Competition

**DOI:** 10.5114/jhk/196719

**Published:** 2025-07-21

**Authors:** Wenping Sun, LianYee Kok, ChenSoon Chee

**Affiliations:** 1 Department of Sports, Nanyang Institute of Technology, Nanyang, China.; 2 Department of Sport Science, Faculty of Applied Sciences, Tunku Abdul Rahman University of Management and Technology, Kuala Lumpur, Malaysia.; 3 Department of Sport Studies, Faculty of Educational Studies, Universiti Putra Malaysia, Kuala Lumpur, Malaysia.

**Keywords:** center, forward, guard, final rankings, team sports

## Abstract

The purpose of this study was to differentiate the attack-defense performance at various playing positions between the top and bottom teams during the Tokyo Olympics men's basketball competition, and to determine the relationship between the attack-defense performance of various positions and the final competition rankings. The rank-sum ratio (RSR) was employed to describe the attack-defense abilities of a total of 144 male players from 12 participating teams, which were divided into three groups according to their playing positions, namely centers (n = 27), forwards (n = 58), and guards (n = 59). Additionally, the independent sample t-test and Spearman Rho Correlation analyses were conducted to evaluate the differences and relationships among the various variables, respectively, at a 0.05 level of significance. The results showed that there were significant differences in points (p = 0.003), the 2-point field goal made percentage (p = 0.035), and defensive rebounds (p = 0.004) at the guard position, and assists (p = 0.047) at the forward position between the top four and the bottom four teams. The attack-defense ranks of the guard position presented high positive correlations (p = 0.000, r = 0.876) with the final competition rankings, while the center (p = 0.017, r = 0.669) and forward positions (p = 0.036, r = 0.608) showed moderate positive correlations. These results may be considered by coaches and players to include targeted training to improve the overall strength of the team.

## Introduction

Basketball is a team sport that utilizes technical and tactical elements, and an organized system of individual and collective tasks that require players to perform different techniques in their playing positions during a game (Perica et al., 2011). Basketball players generally play three traditional positions, namely guards, forwards, and centers, with each position executing different game tasks (Stanković et al., 2022). The center is mainly responsible for defending and rebounding, the forward for attacking and scoring, and the guard is responsible for assists (Nanda and Dimyati, 2019). However, the selection of players to fill these positions depends heavily on his/her body height. Generally, the tallest players will be assigned to play as centers or forwards near the basket, while shorter players usually play the role of guards (Alejandro et al., 2015). Regardless of body stature and also due to the intermittent, dynamic and complex nature of basketball, players are required to perform multiple accelerations, decelerations, jumps, and sprints during the game to complete attacking and defensive tasks (Espasa-Labrador et al., 2023; García et al., 2022a; Vázquez-Guerrero et al., 2019). Therefore, many previous studies have examined professional male basketball players in these common playing positions from different perspectives such as anthropometric characteristics (Mykola et al., 2022), physiological functions (García et al., 2022b; Mykola et al., 2022; Pérez-Chao et al., 2023) and psychological skills (Nanda and Dimyati, 2019).

Technical and tactical skills of basketball have also been investigated previously. It has been suggested that basketball has cooperative-opposition characteristics including phases of attack, defense, and transition in the game (Santos et al., 2018). Attacking and defending are two basic phases in a basketball game that take place sequentially (Stanković et al., 2022) with the basic tenet to attack and defend well. Basketball attack and defense performance indicators include techniques such as rebounds, assists, and steals (Pomeschikova et al., 2015), and the use of statistical measures can quantify these game-related indicators to reflect attacking and defensive ability (Piette et al., 2010). It seems advantageous for coaches to obtain information related to the techniques utilized in specific playing positions that are most conducive for winning games. Therefore, quantitative analysis and evaluation of performance, especially through game-related statistics, seem beneficial when conducting performance analysis in basketball (Zhai et al., 2021). Quantitative analysis has been widely used among coaches to scrutinize game results more convincingly (Sampaio et al., 2004). Such information can help coaches optimize the abilities of players at different positions and strengthen the most important techniques for each position, thus improving the overall ability of the team (Page et al., 2007). In addition, this analysis can be used to determine the most effective players as well as the contribution each player makes to the team as a whole ( Gacek et al., 2024; Pluta et al., 2014).

However, there is a limited number of studies that considered playing positions when documenting differences in game-related statistics. Most of the studies that included playing positions in their analysis considered only few performance indicators (rebounds, 3-point shot percentages, assists, blocks) for distinguishing among centers, forwards, and guards (Fan and Dong, 2017; Sampaio et al., 2006; Zhai et al., 2021). For instance, Sampaio et al. (2006) analyzed game-related statistics among guards, forwards, and centers in three professional men’s basketball leagues run by the National Basketball Association (NBA) (USA), the Asociación de Clubs de Baloncesto (ACB) (Spain), and the Liga de Clubes de Basquetebol (LCB) (Portugal), and found that guards and centers were distinguished in the LCB league by blocks and defensive rebounds, while assists and 3-point field goals were the primary factors in the ACB and the NBA leagues. Another study examining different playing positions of elite women's basketball players found that game-related statistics distinguishing among different positions varied across championships (Zhai et al., 2021). However, the above studies did not integrate the analysis of technique-related performance indicators with attacking and defensive performance, especially the relationship between the attack-defense performance of different playing positions and game outcomes. What is especially scarce is the analysis and comparison of the techniques performed at specific playing positions between top and bottom performing teams during international elite level competitions.

Therefore, the objectives of this study were to analyze and evaluate the attacking and defensive performance at centers, forwards, and guards positions among teams participating in the Tokyo Olympics men's basketball competition, especially focused on the top and bottom four performing teams in order to learn more about the typical and unique traits associated with each playing position among the top basketball teams, differentiate the attack-defense performance indicators between top and bottom four teams, and correlate the attack-defense performance of different playing positions with the final competition rankings. It was hypothesized that there would be significant differences in each attack-defense performance indicator at center, forward, and guard positions between the top four and the bottom four teams. In addition, the attack-defense performance of various playing positions was significantly related to the team’s final competition rankings.

## Methods

### Sample

There was a total of 12 participating teams in the Tokyo Olympics men's basketball competition, and the final rankings are shown in Table 1. Data from the table were obtained from the web page of the *Fédération International de Basketball Amateur* or FIBA (FIBA, 2021), and showed that the sample comprised 144 male players, with 27 centers, 58 forwards, and 59 guards.

**Table 1:** Final team rankings and playing positions during the Tokyo Olympics men's basketball competition.

FR*	Teams	Centers (27)	Forwards (58)	Guards (59)
1	USA	No. 11, No. 13	No. 5, No. 8, No. 9, No. 14	No. 4, No. 6, No. 7, No. 10, No. 12, No. 15
2	France	No. 17, No. 27, No. 28, No. 93	No. 5, No. 7	No. 1, No. 3, No. 4, No. 10, No. 12, No. 21
3	Australia	No. 13, No. 14	No. 6, No. 7, No. 12, No. 15	No. 4, No. 5, No. 8, No. 9, No. 10, No. 11
4	Slovenia	No. 10, No. 27	No. 8, No. 15, No. 30, No. 31, No. 55, No. 77	No. 5, No. 6, No. 7, No. 11
5	Italy	No. 16	No. 8, No. 9, No. 13, No. 17, No. 31, No. 33	No. 0, No. 1, No. 7, No. 24, No. 54
6	Spain	No. 4, No. 13, No. 14, No. 16	No. 3, No. 5, No. 10, No. 20, No. 21	No. 6, No. 9, No. 23
7	Argentina	No. 11, No. 12, No. 83	No. 4, No. 9, No. 14, No. 22, No. 29	No. 7, No. 8, No. 10, No. 17
8	Germany	No. 7	No. 5, No. 6, No. 12, No. 13, No. 19, No. 22, No. 32	No. 0, No. 1, No. 4, No. 42
9	Czech Republic	No. 1, No. 12, No. 15, No. 24	No. 11, No. 17, No. 23	No. 4, No. 8, No. 13, No. 19, No. 25
10	Nigeria	No. 15	No. 0, No. 8, No. 10, No. 33, No. 55	No. 3, No. 11, No. 13, No. 20, No. 22, No. 34
11	Japan	No. 32	No. 8, No. 12, No. 18, No. 23, No. 34, No. 88	No. 2, No. 6, No. 9, No. 14, No. 24
12	Iran	No. 15, No. 23	No. 7, No. 8, No. 14, No. 20, No. 41	No. 3, No. 5, No. 13, No. 17, No. 88

Note. *FR = final ranking of each team during the Tokyo Olympics men’s basketball competition

### Data Collection

A total of 26 matches from the entire competition were used to collect game-related data on the players according to their playing positions. All game-related statistics of each player were collected from the box-score page of the FIBA website (FIBA, 2021). Points (PTS), offensive rebounds (OR), the 3-point field goal made percentage (3P%), the 2-point field goal made percentage (2P%), the free-throw field goal made percentage (FT%), turnovers (To), and assists (As) were included in the attacking performance indicators (Wang, 2017). On the other hand, the defensive performance indicators consisted of defensive rebounds (DR), steals, blocks, and fouls (Hou et al., 2015). For all players, indicators for attacking performance were combined to reflect individual and team attacking ability, while indicators for defensive performance were similarly combined to reflect individual and team overall defensive ability (Hou et al., 2015; Wang, 2017). To ensure the accuracy and consistency of the data, a sub-sample of 8 games was randomly selected from a total of 26 games and observed by two analysts with more than five years of experience in analyzing basketball performance. The results were compared with the data collected from the FIBA official website, and it was found that perfect intra-class correlation coefficients (ICC = 1.0) were obtained for the attacking and defensive performance indicators selected for this study.

The following procedure was used for each team's data collection process: (i) identification of each player according to the playing position (center, forward, or guard) according to the information provided on the FIBA website, (ii) scores for every performance indicator extracted from game-related data of each player, (iii) normalization was performed to categorize each player's game-related data by position. In a basketball game, each player's playing time may be different due to coaching decisions, the player's role (starter or bench player), etc., which leads to different game statistics. However, studies have shown that even when a player's playing time is variable, statistics on a per-minute basis tend to be fairly consistent (Kubatko et al., 2007). Therefore, players' game-related data were normalized in this study on a per-40-min basis because Olympic basketball games last 40 min (Kubatko et al., 2007). The normalized formula for player data is:

OS40 *_A_* = OS *_A_* / MIN *_A_* × 40,

where OS40 *_A_* is normalized to reflect per-40-minute statistics for player *A*, OS *_A_* is original statistics for player *A*, and MIN *_A_* is the number of minutes on the court for player *A*.

### Data Analysis

The attack-defense RSR values of each position for each team were determined by applying the rank-sum ratio (RSR) method. The RSR approach has been widely used for statistical and measurement data analysis in different research fields due to its high capacity and malleability (Wang, 2017). It can represent the average row or column order of a matrix with N rows and M columns, thereby reflecting the comprehensive evaluation of various measurement units and multiple indicators (Wang et al., 2015). Most previous studies (Kang and Yuan, 2017; Li and Sun, 2016) utilized the RSR to examine a team's overall attacking and defensive performance and showed the relationship between attack-defense ability and the final competition rankings. Only one previous study examined associated performance indicators with playing positions (Ji et al., 2021). The RSR is a data process involving rank transformation (Chen et al., 2016). The formula for calculating the RSR is:

RSR = ΣR/(M*n)

where M equals the number of performance indicators, N equals the number of teams, R equals the rank value of each indicator, and ∑R equals the rank sum of all performance indicators for each team. However, for the R value, certain indicators are coded from small to large indices when performance is better when numbers are larger such as for points and rebounds, while indices for turnovers and fouls are inversely assigned, meaning that the higher the value, the worse the rank (Li and Sun, 2016). When teams achieve the same ranks, the mean of these index values is determined (Pan et al., 2016). RSR values range from 0 to 1, and higher numbers denote superior performance or ranking (Li and Sun, 2016; Wang, 2017). Using the 5-level RSR evaluation criteria (Table 2), this study classified attack-defense performance into five categories (A, B, C, D, E) based on RSR values: very strong (RSR values higher than or equal to 0.8), strong (0.60–0.79), moderate (0.40–0.59), weak (0.20–0.39), and very weak (RSR values less than or equal to 0.19). According to the RSR comprehensive evaluation criteria, each team's attacking and defensive capabilities in the same position may then be described. Additionally, the rank and value of performance indicators can be used to highlight the benefits and drawbacks of each team's attack-defense performance at the same positions.

**Table 2:** The RSR comprehensive evaluation criteria.

A	B	C	D	E
≥0.8 Very Strong	0.60–0.79 Strong	0.40–0.59 Moderate	0.20–0.39 Weak	≤0.19 Very Weak

Note. Percentile of the Rank-Sum Ratio (RSR) suggested by Tian Fengdiao

In order to gather data on the common and unique attributes of each position in the top teams and to investigate any disparities in attack-defense performance indicators at the center, forward, and guard positions between the top and the bottom teams, the 12 teams that took part were split into three groups based on their final ranking: the top four, the middle four, and the bottom four. Since there is typically little difference between two groups with similar rankings chosen for comparison, this format of dividing the top and the bottom performing teams with a “middle performing” group is quite frequently used to differentiate performance characteristics among different levels of ability. An independent sample *t*-test was conducted (IBM SPSS Statistics Version 25) to test the differences in attack-defense performance indicators at various positions between the top four and the bottom four teams. Additionally, Spearman rank correlation analysis was conducted at the 0.05 significance level to determine the relationship between the attack-defense performance rankings of different positions and the final competition ranking. All statistical tests utilizing SPSS software were run bootstrapping with 95% confidence intervals.

## Results

### Analysis of the Attack-Defense Performance According to Playing Positions

RSR evaluation focused on three playing positions for all 12 teams in the competition which were the center, forward and guard positions. The RSR comprehensive evaluation of the attack-defense performance for the center position (Table 3) found that the USA had the strongest attack-defense performance among the 12 competing teams, with an RSR value of 0.67 placing it in the “strong” and B category. This was followed by Australia and Slovenia, with RSR values of 0.64 and 0.61, respectively, and all having attained category B. The rest of the teams (France, Italy, Spain, Argentina, Germany, Czech Republic, Nigeria, Japan, and Iran), were ranked at category C, with RSR values of 0.53, 0.59, 0.48, 0.42, 0.59, 0.54, 0.52, 0.41, and 0.51, respectively. The Japanese center position recorded the smallest RSR value, which was the weakest for attack-defense ability among the twelve participating teams.

**Table 3:** The RSR value and grade of the attack-defense performance for the center position.

FR	1	2	3	4	5	6	7	8	9	10	11	12
Teams	USA	France	Australia	Slovenia	Italy	Spain	Argentina	Germany	Czech	Nigeria	Japan	Iran
PTS	18.5	16.9	23.8	20.2	12	14.3	11.3	14	20.8	24	6.9	16.9
R	8	6.5	11	9	3	5	2	4	10	12	1	6.5
2P %	65	64.5	59.6	71.7	25	44.6	54.5	63.6	65.8	85.7	50	45.5
R	9	8	6	11	1	3	5	7	10	12	4	2
3P %	0	50	35.7	38.5	0	28.6	0	35.7	33.3	0	0	50
R	3	11.5	8.5	10	3	6	3	8.5	7	3	3	11.5
FT %	68.8	62.8	73.9	52.9	100	68	75	85.7	56.8	100	50	64
R	7	4	8	2	11.5	6	9	10	3	11.5	1	5
OR	4.4	3	3.2	5.32	4	2.9	4.2	2.8	2.98	5.33	4.1	3.8
R	10	4	5	11	7	2	9	1	3	12	8	6
As	3.2	2.2	4.1	3.1	4	3.98	0.8	3.6	2.3	0.9	0	3.4
R	7	4	12	6	11	10	2	9	5	3	1	8
To	1.76	2.2	1.6	1.8	16	4	2.3	3.2	3.8	8	1.4	4.8
R	10	8	11	9	1	4	7	6	5	2	12	3
DR	7.06	9	4.6	9.2	12	8.9	6	10	5.3	7.1	2.8	8.6
R	5	9	2	10	12	8	4	11	3	6	1	7
Steals	2.9	0.8	1.9	0.37	4	0.36	2.3	1.6	2	0	0	1.7
R	11	5	8	4	12	3	10	6	9	1.5	1.5	7
Blocks	2.6	1.1	0.81	0.2	8	1.4	0.38	0.4	0.83	0	2.8	1.7
R	11	7	5	2	12	8	3	4	6	1	10	9
Fouls	3.82	4.75	3.78	3.9	4	2.9	4.9	2.4	2.8	4.4	2.76	4.8
R	7	3	8	6	5	9	1	12	10	4	11	2
RSR	0.67	0.53	0.64	0.61	0.59	0.48	0.42	0.59	0.54	0.52	0.41	0.51
Grade	B	C	B	B	C	C	C	C	C	C	C	C
Rank	1	7	2	3	4	10	11	4	6	8	12	9

Note. All statistics are per 40 minutes at the center position for each team. FR (The final ranking during Tokyo Olympics men’s basketball game), PTS (Points), 2P % (2-point field goal made percentage), 3P % (3-point field goal made percentage), FT % (Free throws field goal made percentage), OR (Offensive Rebounds), As (Assists), To (Turnovers), DR (Defensive Rebounds)

For the forward position, the RSR comprehensive evaluation of the attack-defense performance among the 12 competing teams (Table 4) found that Slovenia demonstrated the best attack-defense performance, with an RSR of 0.70 placing it in B category. Meanwhile Australia and Italy also ranked at category B, with RSR values of 0.68 and 0.65, respectively. The USA, Nigeria, Spain, France, Japan, Argentina, Germany, and Czech Republic followed with RSR values of 0.59, 0.59, 0.55, 0.50, 0.50, 0.47, 0.47, and 0.46, respectively, all of which fell into the C category. However, Iran's attack-defense ability at the forward position was the weakest among all participating teams, with an RSR of 0.33, and was graded the D category.

**Table 4:** The RSR value and grade of the attack-defense performance for the forward position.

FR	1	2	3	4	5	6	7	8	9	10	11	12
Teams	USA	France	Australia	Slovenia	Italy	Spain	Argentina	Germany	Czech	Nigeria	Japan	Iran
PTS	14.2	12	15.7	21.4	18.6	10.4	17.1	16.6	13.2	19.8	17.5	11.3
R	5	3	6	12	10	1	8	7	4	11	9	2
2P %	67.6	44.1	56.7	64	54.3	66.7	47.9	51.7	52.2	47.5	45.9	51.4
R	12	1	9	10	8	11	4	6	7	3	2	5
3P %	45.5	35	41.4	38.7	36.3	33.3	33.3	38.9	36	51.4	37.5	24
R	11	4	10	8	6	2.5	2.5	9	5	12	7	1
FT %	80	70.6	81.8	72.5	80	100	72.7	86.5	0	51.9	72	50
R	8.5	4	10	6	8.5	12	7	11	1	3	5	2
OR	1.56	1.57	2.9	1.82	2	1.8	2.3	1.7	0.5	1.56	1.73	1.79
R	2.5	4	12	9	10	8	11	5	1	2.5	6	7
As	6	3.6	3.8	5.1	2.29	1.3	2.2	1.7	3.9	2.5	3.1	2.3
R	12	8	9	11	4	1	3	2	10	6	7	5
To	1.7	1.9	2.31	2.8	1.8	1.1	2.3	2.1	2	3.8	3	3.4
R	11	9	5	4	10	12	6	7	8	1	3	2
DR	3.7	6.7	5.3	6.5	5.7	5.03	5.4	5.1	1.6	7.8	5.07	5
R	2	11	7	10	9	4	8	6	1	12	5	3
Steals	2.16	1.1	1.3	1.5	1.27	2.15	0.8	1.2	1.8	3.1	1.29	2
R	11	2	6	7	4	10	1	3	8	12	5	9
Blocks	0.1	1	0.4	0.58	0.8	0.6	0.5	0.2	0.3	1.4	0.54	0
R	2	11	5	8	10	9	6	3	4	12	7	1
Fouls	5.8	3	2.9	3.8	4	4.9	4.1	4.9	2.6	4.4	2.91	3.9
R	1	9	11	8	6	2.5	5	2.5	12	4	10	7
RSR	0.59	0.5	0.68	0.7	0.65	0.55	0.47	0.47	0.46	0.59	0.5	0.33
Grade	C	C	B	B	B	C	C	C	C	C	C	D
Rank	4	7	2	1	3	6	9	9	11	4	7	12

Note. All statistics are per 40 minutes at the forward position for each team. FR (The final ranking during Tokyo Olympics men’s basketball game), PTS (Points), 2P % (2-point field goal made percentage), 3P % (3-point field goal made percentage), FT % (Free throws field goal made percentage), OR (Offensive Rebounds), As (Assists), To (Turnovers), DR (Defensive Rebounds)

When examining the attack-defense performance for the guard position using the RSR comprehensive evaluation (Table 5), the USA had the strongest attack-defense ability among the 12 competing teams, with an RSR value of 0.73. Teams from Australia and France were ranked second and third, respectively, with RSR values of 0.70 and 0.61. These three teams all achieved category B rankings. Teams that ranked further behind were Spain, Italy, Argentina, Slovenia, Japan, Czech Republic, Germany, and Iran, with RSR values of 0.58, 0.56, 0.56, 0.53, 0.52, 0.51, 0.48, and 0.45 respectively, all of which made category C. The attack-defense ability of Nigeria's guards ranked last, with an RSR value of 0.25 (category D).

**Table 5:** The RSR value and grade of the attack-defense performance for the guard position.

FR	1	2	3	4	5	6	7	8	9	10	11	12
Teams	USA	France	Australia	Slovenia	Italy	Spain	Argentina	Germany	Czech	Nigeria	Japan	Iran
PTS	22.5	19.6	18.13	18.1	13.7	27.8	17.2	16.8	13.3	10.2	13.6	14.5
R	11	10	9	8	4	12	7	6	2	1	3	5
2P %	56.3	51.6	47.8	45.5	50	62.7	58.3	49.1	41	27.6	45.5	38.6
R	10	9	6	4.5	8	12	11	7	3	1	4.5	2
3P %	37.5	38.1	39.3	33	22.5	35.7	26.3	39.2	32.3	31.4	32.4	38.9
R	8	9	12	6	1	7	2	11	4	3	5	10
FT %	79.7	85.2	78.1	77.8	89.3	81.8	84.2	73.3	66.7	57.1	100	80
R	6	10	5	4	11	8	9	3	2	1	12	7
OR	1.15	1.1	1.2	1.4	0.76	0.3	0.7	1.3	1.6	0.5	0.8	0.6
R	8	7	9	11	5	1	4	10	12	2	6	3
As	4.6	5.83	5.82	4.38	4.9	6.5	6	4.3	8	4.28	4.4	4.8
R	5	9	8	3	7	11	10	2	12	1	4	6
To	2.2	3.6	2.8	2.3	1.3	3.7	3.59	4.2	2.9	3.75	0.6	3.5
R	10	4	8	9	11	3	5	1	7	2	12	6
DR	5.3	4.4	4.2	4.16	3	3.6	3.9	2.7	3.3	2.8	3.4	2.77
R	12	11	10	9	4	7	8	1	5	3	6	2
Steals	1.69	1.5	2.33	1.3	2.28	0.8	2.32	1.2	2	1.7	0.6	0.62
R	7	6	12	5	10	3	11	4	9	8	1	2
Blocks	1.1	0	0.18	0.22	0	0.16	0	0.21	0	0.7	1	0.15
R	12	2.5	7	9	2.5	6	2.5	8	2.5	10	11	5
Fouls	3.3	4.21	3.4	4.7	2.9	3.9	3.94	2.5	2.94	4.9	4.2	1.8
R	8	3	7	2	10	6	5	11	9	1	4	12
RSR	0.73	0.61	0.7	0.53	0.56	0.58	0.56	0.48	0.51	0.25	0.52	0.45
Grade	B	B	B	C	C	C	C	C	C	D	C	C
Rank	1	3	2	7	5	4	5	10	9	12	8	11

Note. All statistics are per 40 minutes at the guard position for each team. FR (The final ranking during Tokyo Olympics men’s basketball game), PTS (Points), 2P % (2-point field goal made percentage), 3P % (3-point field goal made percentage), FT % (Free throws field goal made percentage), OR (Offensive Rebounds), As (Assists), To (Turnovers), DR (Defensive Rebounds)

### Analysis of the Differences in Attack-Defense Performance between the Top and the Bottom Four Teams According to Playing Positions

When examining the attack-defense performance of the top four and the bottom four teams at the center position (Table 6), there were no significant differences in any of the attacking and defensive performance indicators (*p* > 0.05), and with the bottom four teams performing better in some indicators such as FT%, blocks, and fouls. When comparing attacking and defensive performance indicators at the forward position (Table 6), only assists were found to be significantly different between the top four and the bottom four teams (*p* = 0.047). However, with the exception of performance indicators such as steals, fouls, and blocks, all other performance indicators for the top four teams outperformed the bottom four. At the guard position, the top four teams were significantly different from the bottom four in PTS (*p* = 0.003), 2P% (*p* = 0.035), and defensive rebounds (*p* = 0.004), while the other indicators were not significantly different (Table 6) (*p* > 0.05).

**Table 6:** Differences in attack-defense indicators between the top four and bottom four teams according to playing positions.

Center Position											
	PTS	2P %	3P %	FT %	OR	As	To	DR	Steals	Blocks	Fouls
Top four (x ± S_d_)	19.9 ± 3.0	65.2 ± 5.0	31.1 ± 21.6	64.6 ± 9.0	4.0 ± 1.1	3.2 ± 0.8	1.8 ± 0.3	7.5 ± 2.1	1.5 ± 1.1	1.2 ± 1.0	4.1 ± 0.5
Bottom four (x ± S_d_)	17.2 ± 7.4	61.8 ± 18.2	20.8 ± 25.0	67.7 ± 22.3	4.1 ± 1.0	1.7 ± 1.5	4.5 ± 2.7	6.0 ± 2.5	0.9 ± 1.1	1.3 ± 1.2	3.7 ± 1.1
Difference	2.7	3.4	10.3	−3.1	−0.1	1.5	−2.7	1.5	0.6	−0.1	0.4
T	0.676	0.366	0.619	−0.258	−0.099	1.774	−1.937	0.922	0.725	−0.197	0.643
*P* (2-tailed)	0.524	0.727	0.559	0.805	0.924	0.126	0.101	0.392	0.496	0.850	0.544
Forward Position											
	PTS	2P %	3P %	FT %	OR	As	To	DR	Steals	Blocks	Fouls
Top four (x ± S_d_)	15.8 ± 4.0	58.1 ± 10.4	40.2 ± 4.4	76.2 ± 5.5	2.0 ± 0.6	4.6 ± 1.1	2.2 ± 0.5	5.6 ± 1.4	1.5 ± 0.5	0.5 ± 0.4	3.9 ± 1.3
Bottom four (x ± S_d_)	15.5 ± 3.9	49.3 ± 3.0	37.2 ± 11.2	43.5 ± 30.6	1.4 ± 0.6	3.0 ± 0.7	3.1 ± 0.8	4.9 ± 2.5	2.0 ± 0.8	0.6 ± 0.6	3.5 ± 0.8
Difference	0.3	8.8	3.0	32.7	0.6	1.6	−0.9	0.7	−0.5	−0.1	0.4
T	0.134	1.637	0.485	2.104	1.293	2.498	−1.912	0.472	−1.196	−0.113	0.533
*P* (2-tailed)	0.898	0.153	0.645	0.080	0.244	0.047^*^	0.104	0.653	0.277	0.914	0.613
Guard Position											
	PTS	2P %	3P %	FT %	OR	As	To	DR	Steals	Blocks	Fouls
Top four (x ± S_d_)	19.6 ± 2.1	50.3 ± 4.7	37.0 ± 2.8	80.2 ± 3.4	1.2 ± 1.3	5.2 ± 0.8	2.7 ± 0.6	4.5 ± 0.5	1.7 ± 0.4	0.4 ± 0.5	3.9 ± 0.7
Bottom four (x ± S_d_)	12.9 ± 1.9	38.2 ± 7.6	33.8 ± 3.5	76.0 ± 18.6	0.9 ± 0.5	5.4 ± 1.8	2.7 ± 1.4	3.1 ± 0.3	1.2 ± 0.7	0.5 ± 0.5	3.5 ± 1.4
Difference	6.7	12.1	3.2	4.2	0.3	−0.2	0.0	1.4	0.5	-0.1	0.4
T	4.794	2.708	1.458	0.450	1.308	−0.220	0.048	4.617	1.115	-0.258	0.580
*P* (2-tailed)	0.003^**^	0.035^*^	0.195	0.669	0.239	0.833	0.964	0.004^**^	0.308	0.805	0.583

Note. ** p < 0.01, with a very significant difference, * p < 0.05, with a significant difference

### Relationship between the Attack-Defense RSR Ranks of Different Positions and the Final Rankings

The Spearman Rho correlation was employed to examine the correlation between the attack-defense RSR ranks of centers, forwards, and guards and the final rankings. The teams' final competition rankings were the dependent variables, and the RSR rankings of the guard, center, and forward positions among the 12 competing teams were the independent variables. Table 7 shows the correlation coefficients between the final rankings and the attack-defense RSR ranks of various positions. The center (*p* = 0.017, *r* = 0.669), forward (*p* = 0.036, *r* = 0.608), and guard positions (*p* = 0.001, *r* = 0.876) all indicated a significant and positive relationship with the final rankings. However, based on the Guildford Rule of Thumb, the guard position had a high association with the final rankings, whereas the center and forward positions showed a moderate correlation.

**Table 7:** Correlation between the attack-defense RSR ranks of different positions and the final ranking.

	Centers	Forwards	Guards
Spearman’s rho	0.669*	0.608*	0.876**
Sig.(2-tailed)	0.017	0.036	0.000
N	12	12	12

Note. * Correlation is significant at the 0.05 level (2-tailed), ** Correlation is significant at the 0.01 level (2-tailed)

## Discussion

The main findings of the present study are that the teams with the best attack-defense abilities at both the center and guard positions during the Tokyo Olympics men's basketball competition were the USA, while Slovenia had the best attack-defense performance at the forward position. Between the top and the bottom ranked four teams, there were no significant differences in any of the attack-defense performance indicators at the center position. However, the top four teams outperformed the bottom four teams in PTS, 2P%, and defensive rebounds at the guard position, as well as assists at the forward position, showing significant differences. Additionally, the results indicated that the attack-defense RSR ranks of the guard position had the highest correlation with the final competition rankings compared to the other two positions. Also, the attack-defense ability at the guard position seemed to be an important factor for differentiating between the top and the bottom ranked teams during this competition.

Previous studies analyzing basketball performances have also proposed that the actual performance of each player determines the team's results in basketball competition, usually represented by game-related performance indicators such as the field goal percentage, rebounds, and steals (Fan and Dong, 2017; Kokanauskas et al., 2021; Sampaio et al., 2006; Zhai et al., 2021). For example, one study was found that scrutinized the performance profile of youth basketball players in European youth championships (Kokanauskas et al., 2021) with results showing that players from the top four teams performed significantly better in terms of field goals scored, the field goal percentage, 2-point shots scored, offensive rebounds, defensive rebounds, assists, fouls, turnovers, and blocks compared to players from the bottom four teams. However, the results from youth basketball did not agree with those found here that analyzed and compared variables based on playing positions from the top and bottom performing teams during an international elite level competition. Significant differences in PTS, 2P%, and defensive rebounds at the guard position were found between the top four and the bottom four teams, as well as assists at the forward position. Based on previous literature, the reason for the inconsistency between the two results may be attributed to relative age effects (RAEs) (Ibáñez et al., 2018). Studies on the RAE on different positions in senior basketball have shown that the RAE impacts more prominently the guard position (Ibáñez et al., 2018), as guards decline in physical fitness with age, yet the efficiency index and technical performance remain stable or even improve (Kalén et al., 2021). Evidence suggests that most elite basketball players began basketball-specific training after the age of 11 (Leite and Sampaio, 2012), and guards differed from other positions in that their continuity in high-level competition relied more on athletic abilities and skills than on anthropometric measurements (Ibáñez et al., 2018). Thus, it can be argued that relatively older guards implied more athleticism and game experience, they were able to acquire performance skills more quickly, and had higher scoring and 2-point shooting percentages (Ibáñez et al., 2018; Kalén et al., 2021). In the study by Kokanauskas et al. (2021) on European youth championships, players were all at the same age stage, such as U-16, U-18, or U-20, and there was no age gap. However, each team was composed of players of different ages in the Olympic basketball tournament. The average age of the starting guards for the top four teams in this study was 32, compared to 28 for the bottom four teams. Therefore, the age gap at the guard position between the two groups may be an important factor leading to the results of this study.

Additionally, Page et al. (2007) examined game-related statistics for different positional players in National Basketball Association (NBA) leagues and found that the shooting percentage at the guard position had a greater impact on the outcome of the game compared to centers and forwards, which is consistent with the findings of this study. The reason for this is that the further you are from the basket, the more difficult it is to score (Page et al., 2007). Therefore, it was not surprising that guard players who shoot a much higher percentage than their opponents at the same position may lead to better team results. Furthermore, one study on the NBA analyzed the impact of different teams' performance at similar playing positions on the outcome of a game (Zhang et al., 2019), and reported that guards from the winning team made more steals and fewer fouls, and centers made more defensive rebounds and blocks during home games, while centers of the winning team got more assists and steals, and forwards got more free throws in away games. Another study on the anthropometric attributes and playing experience of NBA players found that players from weaker teams made the fewest passes, implying a lack of assisting ability and a sense of teamwork, while several of the superpowered teams that made it to the finals highlighted players with the fewest turnovers (Zhang et al., 2018). However, the results of this study were a little different from those of the two studies mentioned above. First of all, there is no distinction between home and away in the Olympic Games, thus performance of players cannot be affected by objective situations like this one. Second, the lack of assisting ability of the weaker team in this study was only reflected in the forward position, while, although the stronger team had fewer turnovers at all positions than the weaker team, it was not sufficient to be an important factor in differentiating between the two. The reason for this may be that as modern basketball continues to evolve, high-level teams focus more and more on taller and quicker players, thus guard players may approach the body height of forward players rather than the traditional smaller ones (Ji et al., 2022; te Wierike et al., 2014). However, some Asian teams such as Japan and Iran in the bottom four teams of this study still continue the tradition of small guards, which may be the main reason why the guard position is not dominant in grabbing rebounds when they face high-level teams.

In basketball, each position has specific requirements and characteristics of athletic ability, as well as technical and tactical mastery to meet the goals of the basketball game and implement plans to achieve them during the game (Stanković et al., 2022). For instance, the guard is the player who organizes the team's offense and needs to have good court vision and passing skills (Kokanauskas et al., 2021). Additionally, guards spend most of their time away from the basket, thus a good shooting ability is one of the skills that must be possessed (Ibáñez et al., 2018). The results of this study confirm this detail, as points and the 2-point field goal made percentage at the guard position showed a very significant difference between the top and the bottom teams. However, there were no significant differences in attack-defense indicators between the top and the bottom teams at the center position, with the forward position reflected only in assists. This corroborated the finding of this study that the guard position was more strongly associated with final competition rankings than the center and forward positions.

Besides that, the competition in world basketball is not only a contest of overall ability of a team, but also a competition of the ability of players in corresponding positions (Fan and Dong, 2017). With the development of modern basketball, athletes are becoming more complete in their athletic abilities, showing versatility and being able to play multiple positions and perform different duties in different scenarios of the game (Fan and Dong, 2017; Ji et al., 2022). In other words, the more outstanding the players, the higher their versatility (Rangel et al., 2019). Therefore, these are indicators that the traditional duties of centers, forwards, and guards have gradually evolved. For example, some centers can shoot from outside the 3-point arc, move out of the paint to assist teammates in scoring, while being able to play the role of forwards and even guards (Ji et al., 2022). These characteristics of players were established through analysis of different basketball leagues such as the Brazilian National League (NBL) (Rangel et al., 2019), the Croatian First League (Dežman et al., 2001), and the NBA competition (Ji et al., 2022). Similar results were found in the present study. Generally, a traditional forward player's primary role is to assist inside players in contesting rebounds and scoring, and a guard player's primary role is to assist teammates in scoring and shooting three-pointers (Ji et al., 2022; Kokanauskas et al., 2021). However, as indicated by this study, assists for the forward position and defensive rebounds for the guard position have also become significant variables in distinguishing between the strong and the weak teams, again highlighting the necessity for versatility in the modern basketball player. Nonetheless, the versatility of players does not mean the disappearance of positions, yet the pursuit of a higher and more comprehensive competition ability based on players’ excellent skills in traditional playing positions (Zhou, 2012).

It is important to recognize several limitations when interpreting the study's findings. First of all, the small sample size was one of the limitations of this study, which only referred to the relevant game data from the Tokyo Olympics men's basketball tournament. Secondly, disparities in the fundamental attributes (e.g., physical and physiological traits) of players between the top and the bottom teams might also lead to different performances on attack and defense. However, this aspect was not considered in this study. Therefore, the analysis of multiple elite-level international basketball competitions can provide a more complete picture of the attack-defense ability at different positions in future studies.

## Conclusions

The results of this study indicate that there were significant positive correlations between attack-defense performance and the final team rankings for each of the center, forward, and guard positions in the Tokyo Olympics men's basketball tournament, with the guard position having the strongest correlation of the three. The common characteristics presented by the top men's basketball teams included a strong ability in points, the 2-point field goal made percentage, defensive rebounding at the guard position, and assisting at the forward position. Furthermore, the versatility of players at different playing positions was still a trend in basketball. Coaches can conduct some targeted training to improve the strength of players at various positions. Additionally, this study provides some reference for coaches to select players for different positions based on the level of technical indicators.

## Practical Implications

The findings of this study will assist national men's basketball teams competing in the Tokyo Olympics in recognizing the attack-defense strengths and weaknesses of various positions and providing technical references to help them prepare for future international competitions. Especially for lower-ranked teams, the focus should be on developing the guard position's attack-defense ability, as evidenced by an increase in the shooting percentage and awareness of grabbing defensive rebounds. When selecting guards, it is advisable to go for taller and speedier athletes. Furthermore, forward players should aim to increase their teamwork awareness and assist skills.
